# Gene-Environment Interactions in Attention-Deficit/Hyperactivity Disorder Symptom Dimensions: The Role of Unhealthy Food Habits

**DOI:** 10.3390/genes13010047

**Published:** 2021-12-24

**Authors:** Lin Li, Mark J. Taylor, Katarina Bälter, Tian Xie, Berit Skretting Solberg, Jan Haavik, Alejandro Arias Vásquez, Catharina A. Hartman, Henrik Larsson

**Affiliations:** 1 School of Medical Sciences, Örebro University, 70172 Örebro, Sweden; Henrik.Larsson@oru.se; 2 Department of Medical Epidemiology and Biostatistics, Karolinska Institutet, 17165 Stockholm, Sweden; mark.taylor@ki.se (M.J.T.); katarina.balter@mdh.se (K.B.); 3 Department of Public Health Sciences, Mälardalen University, 72220 Västerås, Sweden; 4 Interdisciplinary Center Psychopathology and Emotion Regulation (ICPE), Department of Psychiatry, University Medical Center Groningen, University of Groningen, 9713 Groningen, The Netherlands; t.xie@umcg.nl (T.X.); c.hartman@accare.nl (C.A.H.); 5 Department of Biomedicine, University of Bergen, 7804 Bergen, Norway; Berit.Skretting-Solberg@uib.no (B.S.S.); jan.haavik@uib.no (J.H.); 6 Child- and Adolescent Psychiatric Outpatient Unit, Hospital Betanien, 5012 Bergen, Norway; 7 Bergen Center of Brain Plasticity, Division of Psychiatry, Haukeland University Hospital, 5009 Bergen, Norway; 8 Departments of Psychiatry & Human Genetics, Donders Institute for Brain, Cognition, and Behavior, Radboud University Medical Center, 6525 Nijmegen, The Netherlands; Alejandro.AriasVasquez@radboudumc.nl

**Keywords:** gene-environment interactions, ADHD, food habits, twin study

## Abstract

*Background:* Dietary habits were investigated as environmental risk factors for Attention-Deficit/Hyperactivity Disorder (ADHD). However, no previous studies explored the effects of dietary factors on modifying the role of genetic factors on ADHD. *Methods:* Based on a Swedish population-based twin study with 1518 twin pairs aged 20–47 years, we tested whether the importance of genetic and environmental effects on ADHD varied as a function of dietary habits. Self-reported dietary habits and ADHD symptoms were collected. Twin methods were used to test the degree to which high-sugar and unhealthy food intake moderated the genetic and environmental influences on ADHD symptoms. *Results:* In middle-aged adults, genetic influences on inattention symptoms were statistically significantly higher among individuals with higher levels of high-sugar (45%, 95%CI: 25–54%) and unhealthy food intake (51%, 95%CI: 31–60%), compared with those with lower levels of consumption of high-sugar (36%, 95%CI: 25–47%) and unhealthy foods (30%, 95%CI: 20–41%). Similar patterns were also found for the associations between hyperactivity/impulsivity and high-sugar/unhealthy food intake, even though the moderation effects were not statistically significant. *Conclusion* The present study suggests that genetic factors play a more prominent role in individual differences of ADHD symptoms in the presence of the high consumption of sugar and unhealthy foods. Future longitudinal studies with multiple assessments of ADHD and dietary habits are needed to replicate our findings.

## 1. Introduction

Attention-deficit/hyperactivity disorder (ADHD) is a common neurodevelopmental disorder characterized by developmentally excessive inattention–disorganization and hyperactivity–impulsivity symptoms [1]. Although onset is in early childhood, ADHD commonly persists into adulthood [2,3]. ADHD is associated with many adverse outcomes across development, exerting a considerable burden on physical and mental health [1,4]. Both genetic and environmental factors contribute to the risk of ADHD. Twin research indicates moderate-to-large genetic contributions; the estimated heritability (that is, the proportion of phenotypic variation attributable to genetic variation) for ADHD is 30–80%, depending on the age, the assessment method used, and the informants [5,6,7]. However, so far, molecular genetic studies identified only a small number of genetic variants that may be directly involved in the aetiology of ADHD, which also explain very little of the variance in ADHD [8,9]. The mismatch between the estimated heritability of ADHD from quantitative genetic research and genome-wide association studies (GWAS), together with the multifactorial and polygenetic nature of ADHD [10,11], suggests that, in addition to individual genes and environmental factors, interactions of several genes with each other and with the environment may also influence variations in the phenotype.

A key framework for understanding the complex contribution of both genetic and environmental influences on ADHD is gene–environment interactions (G × E), that is, the importance of genetic factors may be dependent on key environmental factors [12]. Studies demonstrated that environmental exposures can modify gene expression through epigenetic mechanisms, for example, in psychiatric disorders [13]. The identification of G × E effects is important to advance the understanding of genetic and clinical heterogeneity of ADHD symptoms over time [14]. To date, the most widely accepted model of G × E influences on psychopathology is the diathesis–stress model, in which genes and stressful environments exert risk synergistically. That is, environments may moderate the effects of genetic factors in a phenotype by magnifying the likelihood that genetically vulnerable individuals will develop negative outcomes, resulting in higher genetic variance in adverse environments but lower genetic variance in beneficial environments [14,15,16]. Alternatively, interactions can follow the so-called bioecological model, in which genetic influences on the condition are thought to be more influential in the absence of adversity, resulting in higher heritability in low-risk environments [15,17].

As suggested by available studies, genetic risk variants associated with ADHD seem to have a stronger impact on the presence of unfavourable environmental conditions [18]. Emerging evidence indicates that diet may modify the genetic liability underlying ADHD. Previous research found a direct association between the Western/unhealthy diet (characterized by high consumption of refined sugar and saturated fat but low consumption of fruits and vegetables) [7,19,20] and a low adherence to a Mediterranean diet with elevated ADHD symptoms [21]. Higher sugar intake was found to be associated with more ADHD symptoms in children over 7 years old, and adults with elevated levels of ADHD symptoms were found to be associated with a higher intake of high-sugar food, meat and fat, as well as a lower intake of fruits and vegetables [7]. Findings from a twin study further indicated that genetic factors could only explain a minor part of this association [7].

Several studies used a candidate gene (e.g., 480-bp DAT1, LPHN3 rs1868790) approach to explore G × E interactions in ADHD [22,23,24]. However, studies of candidate G × E interactions are problematic as a priori chosen genetic risk variants rarely replicate in other samples [25,26]. In contrast, latent G × E interactions tested in twin studies examined the aggregate effects of genetic variation rather than the effect of specific genetic variants. Such studies are needed to provide directions for genome-wide G × E studies and G × E studies based on polygenic risk scores [27]. Available twin G × E interaction studies in ADHD focused on family environmental risk factors. For example, Pennington et al. found diathesis–stress effects for socioeconomic status (SES), with higher heritability estimates in disadvantaged households [15]. In contrast, Nikolas et al. reported bioecological interactions for ADHD and parental behaviours, with stronger genetic effects found in some beneficial environments (e.g., lower levels of parental conflict [28] and more parental involvement [29]). Another study suggested that SES moderated the heritability of inattention or hyperactive/impulsive symptoms [30]. However, no previous study explored the effects of dietary factors on modifying the role of genetic effects on ADHD.

The purpose of the present study was to study how dietary habits influence the magnitude of genetic and environmental contributions to ADHD in adults, considering the inattentive and hyperactive/impulsive symptom dimensions separately. Based on previous literature, we hypothesized that genetic factors associated with ADHD were more likely to cause the disorder among adults with unhealthier food habits, as compared to adults with healthier food habits.

## 2. Methods

### 2.1. Participants

Data were collected from participants in the Study of Twin Adults: Genes and Environment (STAGE), which only includes twins born in Sweden between 1959 and 1985 [31]. In the present study, we used data from participants that filled out a web-based survey regarding their sociodemographic information, lifestyle, food habits, mental, and physical health from 2005 to 2006. Standard and validated questions through genotyping were used to assess physical similarity in order to establish zygosity [7]. The study sample comprised 1518 twin pairs with completed information on all study variables.

### 2.2. Measures

*Self-reported ADHD symptoms*: were obtained via an 18-item questionnaire derived from the Diagnostic and Statistical Manual of Mental Disorders, fourth edition (DSM-IV), with two subscales reflecting the inattentive and hyperactive/impulsive symptom dimensions [32]. Both inattention and hyperactivity/impulsivity showed a good internal consistency, and Cronbach’s α was 0.79 and 0.77, respectively [32]. As suggested in previous twin and genetic studies, ADHD is best viewed as the extreme of a continuous trait that is genetically linked with ADHD [33], in this study, ADHD symptoms were measured on a continuum rather than collapsed to a dichotomous diagnosis.

*Food habits:* were assessed by a food frequency questionnaire (FFQ) with 94 food items. For each food item, the participants reported their average consumption frequency over the past year by selecting one of the following frequencies: never, 1–3 times/month, 1–2 times/week, 3–4 times/week, 5–6 times/week, 1 time/day, 2 times/day, 3 times/day. The frequency for each food item was converted into number of servings per day. Consistent with a previous study [7], high-sugar foods included cookies, crackers, cake, chocolate, candy, ice cream, jam, berry cream, juice, energy drink, honey, and ketchup. Unhealthy food included pizza, sausage, fried potato, French fries, minced meat/meatball, pork, beef, cookies, crackers, cake, chocolate, candy, ice cream, jam, berry cream, juice, energy drink, hamburgers, chips, dressing, mayonnaise, cream, and ketchup. Their overall intake frequencies, respectively, were thereafter summed up and categorized into tertiles; (1) high-sugar food: low (≤1.56 servings/day), middle (1.56 to 2.90 servings/day) and high consumption (≥2.90 servings/day); (2) unhealthy food: low (≤2.61 servings/day), middle (2.61 to 4.00 servings/day) and high consumption (≥4.00 servings/day).

### 2.3. Statistical Analyses

Twin data can be used to estimate the genetic and environmental contributions to variation in a trait by comparing the similarity between monozygotic twins (MZ, who share 100% of their segregating genes) and dizygotic twins (DZ, who share approximately 50% of their segregating genes). In the additive model, total phenotypic variance of a trait can be broken down into three components: A—additive genetic influences, or narrow-sense heritability; C—shared environmental influences, which are common to both twins in a pair and make them similar; and E—non-shared environmental influences, which impact the twins individually, and also include random measurement errors [34].

Intra-class correlations were calculated for MZ and DZ twins by ADHD symptom dimensions and each dietary habit group. For our primary analyses, we first standardized the log-transformed ADHD symptoms to have means of 0 and standard deviations of 1. Age and sex were included as covariates on the mean of the phenotype. Second, we estimated the variance of ADHD by A, C and E components. We then excluded C component to test if the AE model was not statistically significantly different from the full ACE model (i.e., the shared environmental component C was not significant), if so, AE models were used in further analyses.

**Figure 1:**
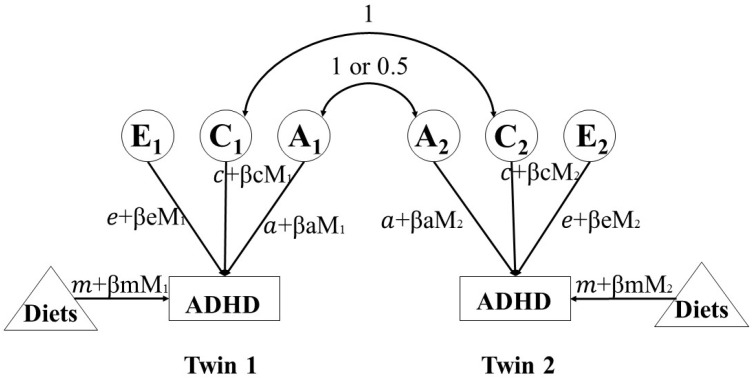
Path diagram for gene–environment interaction model. A: additive genetic influences; C: shared environmental influences; E: non-shared environmental influences; A is correlated to 1 between monozygotic twins (MZ) and 0.5 between dizygotic twins (DZ), respectively; C is correlated to 1 for both MZ and DZ twins; a, c, and e: unmoderated components; βa, βc and βe: moderated components of a, c, and e; m: grand mean; βm: effect of moderator (diets). ADHD: Attention-Deficit/Hyperactivity Disorder.

Next, we expanded our analyses by evaluating whether dietary habits moderated the aetiology of ADHD using the ‘extended univariate G × E’ model (Figure 1), which estimates how variance in phenotypes changes across levels of a measured environmental moderator by including its effect on the paths for each component [35]. G × E is demonstrated by moderation of the three variance components (A, C, and E). Depending on the direction of the moderation effects, a gene–environment interaction could then manifest as an increase or decrease in the heritability of a trait. For example, if the moderator variable decreased genetic variance while increasing shared or non-shared environmental variance, the proportion of total variance explained by genetics would decrease.

To test the gene–environment interaction hypotheses, we determined whether the moderation paths significantly improved the fit of the full ACE/AE moderation model using a negative two times the log-likelihood value (−2lnL) statistic. First, a global test of moderation was conducted by constraining the moderation effects on all of the components to be zero. Second, to test whether the moderation was driven by specific pathways, we then fitted a series of nested models, dropping moderation of the mean components, the variance components and all moderation separately and assessing the change in fit. If dropping moderation paths statistically and significantly reduced model fit (indicated by a significant chi-square χ2 statistic), then the result supported the hypothesis that the environmental variable moderated the heritability of ADHD symptoms. Otherwise, if the model fit was not significantly worse with moderator paths excluded, then dietary habits did not modify the heritability of ADHD symptoms [30].

All analyses were performed by using the structural equation modelling in the statistical software R version 3.6.1, with the OpenMx package (2.14.11).

## 3. Results

Descriptive statistics for the study population are presented in Table 1. The average age (mean ± std.) of the 3036 individuals was 33.17 ± 7.76 years old, the majority were female (67.56%) and had higher levels of socioeconomic status (SES) (74.20%). A total of 47.36% of the individuals were monozygotic twins and 52.64% were dizygotic twins with the same or opposite sex. ADHD symptom scores were significantly higher in the group with a high consumption of high-sugar and unhealthy foods, compared to the groups with a low and average consumption, indicating that the higher consumption of high-sugar and unhealthy foods was associated with more ADHD symptoms. The univariate model-fitting results are displayed in Supplementary Material Table S1. AE models provided the best fit to the data on ADHD symptom dimensions; therefore, we used only AE models in further analyses. Twin correlations and basic estimates of heritability are presented in Table 2. We found that both ADHD symptoms and food habits showed a moderate genetic influence. For ADHD symptoms, genetic and non-shared environmental factors explained 38–41% and 59–62% of the variance, respectively. The corresponding estimates for food habits were 35–37% and 63–65%.

**Figure 2:**
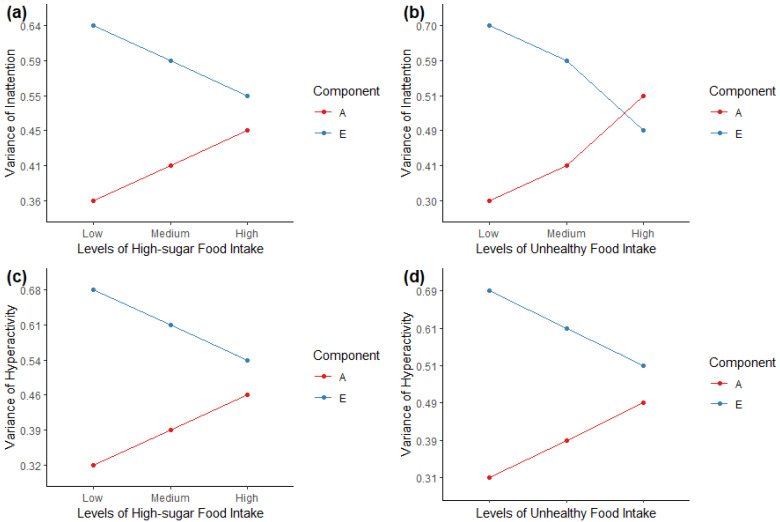
Genetic (A) and non-shared environmental (E) variance of ADHD symptoms change with different levels of high-sugar and unhealthy food intake. (**a**) Full moderation model for inattention and high-sugar food; (**b**) Full moderation model for inattention and unhealthy food; (**c**) Full moderation model for hyperactivity/impulsivity and high-sugar food; (**d**) Full moderation model for hyperactivity/impulsivity and unhealthy food.

Next, to address our main research question, we tested the significance of the moderation between ADHD symptoms and dietary habits by dropping parameters from the best-fitting AE models. Table 3 shows the fit statistics for the full moderation models, followed by the fit statistics after dropping the moderation of the mean and variance components, and then the no-moderation model (with all moderation paths dropped). We found that the full AE model fitted best for inattention and diets, while dropping the variance moderation model fitted best for hyperactivity/impulsivity and diets. We additionally report separately the path estimates for the variance components of the full AE models with moderation effects for inattention and hyperactivity/impulsivity symptoms in Table S2. Overall, we found similar patterns among the associations between ADHD symptoms and dietary habits. The mean and total variance of inattention and hyperactivity/impulsivity was higher with an increased level of high-sugar and unhealthy food intake. We then plotted the variance components, A and E, as a function of dietary habits. Figure 2 illustrates that, at higher levels of high-sugar or unhealthy food intake, genetic components had stronger influences on ADHD symptoms. Specifically, for inattention, the influence of genetic effects on inattention was higher among those with higher consumptions of high-sugar food (genetic factors accounted for 45% of the variance in inattention at the highest level but 36% at the lowest level) and unhealthy food (genetic factors accounted for 51% at the highest level and 30% at the lowest level), while the non-shared environmental effects were lower with the increased levels of high-sugar and unhealthy food intake. Similar patterns were also found in the associations between hyperactivity/impulsivity and high-sugar/unhealthy dietary intake, even though their moderation effects were not statistically significant (Table 3).

## 4. Discussion

To our knowledge, this is the first twin study to test whether two dietary habits, high-sugar food and unhealthy food, moderate the heritability of ADHD symptoms. We found that in middle-aged adults, dietary habits significantly moderated genetic and non-shared environmental contributions to ADHD symptomatology. Specifically, genetic influences on ADHD symptoms increased at higher levels of high-sugar and unhealthy food intake but were lower when consuming less of these diets. By contrast, non-shared environmental contributions to ADHD symptoms were highest at lower levels of high-sugar and unhealthy food intake and decreased as these dietary consumptions increased. Such findings thus serve to not only illuminate the origins of ADHD, but also provide important empirical support the diathesis–stress model of G × E (with the stressors being high-sugar and unhealthy foods).

There are several potential explanations for how dietary factors moderate the influence of genetic and environmental effects on ADHD. Firstly, the field of nutrigenomics has yielded valuable information on how dietary and nutrition influences an individual’s genetic composition and gene expression [36,37]. Results from nutrigenomics studies suggested that a high-sugar diet remodelled fundamental aspects of gene regulation (such as differential DNA methylation, differential gene expression, alternative splicing, and implications for microRNA alterations) that have the potential to impact pathogenesis of metabolic and psychiatric disorders [38]. Secondly, there is increasing evidence suggesting that the microbiome–gut–brain axis is involved in the aetiology of ADHD [39]. Diet rapidly and reproducibly alters the human gut microbiome [40], which then plays an important role in regulating the availability of circulating tryptophan, altering the expression of some CNS receptors, thereby enabling them to directly influence brain excitability and function as well as to exert epigenetic control of gene expression [41]. Therefore, diet could potentially modify the complicated genome–microbiome associations in ADHD. Third, animal studies indicated that regular sugar consumption could change gene expression and the availability of dopamine receptors in brain regions related to reward sensitivity and motivation [42]. Furthermore, many ADHD-related genes are highly expressed in the hypothalamus, which is a key regulator of food intake [43]. Therefore, it is possible that both the gene expression and the function of dopamine can be altered in certain areas of the brain from habitual intake of high-sugar or unhealthy diets, which subsequently increases the risk of ADHD. Future molecular genetic insights into the effects of gene–environment interactions would improve our understanding of the aetiology of ADHD.

Some limitations of the study warrant discussion. Firstly, the current sample was underpowered for examining G × E effects separately by sex and age. Even though we found stable phenotypic and genetic associations between ADHD symptoms and dietary habits across age, sex and SES groups, and we also adjusted for age and sex in the G × E modelling, the future examination of sex and age differences in aetiological mechanisms, including G × E effects, remains important. Second, we examined ADHD symptoms dimensionally by using self-reported data. Given that self-reports among adults typically underestimate the presence of ADHD symptoms and also generate lower heritability estimates compared to reports from other informants (e.g., self- and parent-ratings combined) [5], future studies of gene–environment interactions need to use alternative measurement methods [5]. Our findings may not necessarily generalize to clinically diagnosed ADHD. However, examining inattention and hyperactivity–impulsivity separately could provide additional clues regarding the heterogeneity of aetiological mechanisms in ADHD [44]. Further, food intake was self-reported and may suffer from potential misclassification bias. Additionally, diet was only assessed once in this cross-sectional study, which did not take into account changes over time. Finally, although the findings suggested the presence of a G × E interaction between ADHD symptoms and dietary habits, they may be partly explained by G × E correlation, which reflects genetic differences in the exposure to particular environments [35,45]. As genetic correlations between ADHD symptoms and high-sugar/unhealthy food intake were reported to be relatively weak, ranging from 0.05 to 0.16 [7], the presence of G × E correlation may not fully explain the observed effects of G × E interactions [46]. Models (extended bivariate G × E models) were developed to take into account the G × E correlation effects [35], but such models suffer from a series of methodological problems [35,47] and larger sample sizes are needed than those we currently have at our disposal. Thus, we restricted our analyses to more stable univariate models. Previous studies also reported robust results when extending univariate to bivariate G × E models [16].

## 5. Conclusions

The present study showed that the heritability of ADHD symptoms was higher with a high consumption of high-sugar and unhealthy foods, a finding that supports the diathesis–stress model. The finding provides further insights into a better understanding of mechanisms underlying ADHD, as well as directions for genome-wide G × E studies. Future longitudinal studies with multiple assessments of ADHD and dietary habits are still needed to replicate our findings.

## Figures and Tables

**Table 1:** Means and standard deviations (S.D.) for ADHD scales and intake frequency of high-sugar food and unhealthy, respectively, by age and sex group.

	Total	Consumption of High-Sugar Food			Consumption of Unhealthy Food		
	*n* = 3036	Low Level (*n* = 1007)	Middle Level (*n* = 984)	High Level (*n* = 1045)	Low level (*n* = 995)	Middle Level (*n* = 1010)	High Level (*n* = 1031)
Age	33.17 ± 7.76	33.87 ± 7.81	33.40 ± 7.73	32.29 ± 7.63 *	33.40 ± 7.86	33.51 ± 7.68	32.63 ± 7.69 *
Sex							
Male	985 (32.44)	255 (25.32)	301 (30.59)	429 (41.05) *	234 (23.52)	315 (31.19)	436 (42.29) *
Female	2051 (67.56)	752 (74.68)	683 (69.41)	616 (58.95)	761 (76.48)	695 (68.81)	595 (57.71)
SES							
Low level	573 (25.80)	165 (21.57)	180 (24.46)	228 (31.67) *	163 (22.18)	189 (24.74)	221 (30.61) *
High Level	1648 (74.20)	600 (78.43)	556 (75.54)	492 (68.33)	572 (77.82)	575 (75.26)	501 (69.39)
Zygosity							
MZ	1438 (47.36)	464 (46.08)	468 (47.56)	506 (48.42)	454 (45.63)	491 (48.61)	493 (47.82)
DZ	1598 (52.64)	543 (53.92)	516 (52.44)	539 (51.58)	541 (54.37)	519 (51.39)	538 (52.18)
							
Inattention	2.05 ± 2.11	1.79 ± 1.95	2.08 ± 2.10	2.28 ± 2.24 *	1.89 ± 2.00	1.97 ± 2.06	2.30 ± 2.23 *
Hyperactivity/impulsivity	2.08 ± 2.14	1.94 ± 2.05	2.09 ± 2.18	2.21 ± 2.19 *	1.98 ± 2.09	1.98 ± 2.11	2.28 ± 2.20*

* *p*-value < 0.05 for mean difference based on Linear mixed effect model; for proportion difference based on chis-q test; SES: socioeconomic status; MZ: monozygotic twins; DZ: dizygotic twins.

**Table 2:** Intra-class correlations and estimates from univariate twin models with 95% confidence intervals for ADHD trait dimensions and dietary variables.

	Intraclass Correlations		Parameter Estimates	
	MZ	DZ	A	E
Inattention	0.41 (0.35, 0.47)	0.11 (0.04, 0.18)	0.41 (0.36, 0.47)	0.59 (0.53, 0.64)
Hyperactivity/impulsivity	0.42 (0.36, 0.48)	0.11 (0.04, 0.18)	0.38 (0.32, 0.43)	0.62 (0.57, 0.68)
High-sugar food	0.39 (0.33, 0.45)	0.16 (0.09, 0.22)	0.37(0.31, 0.42)	0.63 (0.58, 0.69)
Unhealthy dietary pattern	0.39 (0.33, 0.45)	0.17 (0.10, 0.23)	0.35 (0.29, 0.41)	0.65 (0.59, 0.71)

*p*-value < 0.05 for all presented analyses. MZ: monozygotic twins; DZ: dizygotic twins; A: additive genetic influences, or narrow-sense heritability; E: non-shared environmental influences.

**Table 3:** Model-fitting results of moderation models with 95% confidence intervals and dietary variables.

		Fit of Model Compared to Saturated Model						
		EP	−2lnL	df	χ^2^	∆df	*p*-Value	AIC
IA: High-sugar dietary intake moderation model								
	Full AE model	6	12,842.48	2999	0.29	1	0.59	6844.47
	Drop moderation of the mean components	6	14,204.83	2999	1362.64	1	<0.001	8206.83
	Drop moderation of the variance components	4	12,858.99	3001	16.80	3	0.001	6856.99
	Drop all moderation	3	14,238.42	3002	1396.23	4	<0.001	8234.42
IA: Unhealthy dietary intake moderation model								
	Full AE model	6	12,857.35	3003	0.21	1	0.65	6851.35
	Drop moderation of the mean components	5	14,242.17	3004	1384.82	1	<0.001	8234.17
	Drop moderation of the variance components	3	12,873.33	3006	15.97	3	0.001	6861.33
	Drop all moderation	2	14,314.55	3007	1457.19	4	<0.001	8300.55
HI: High-sugar dietary intake moderation model								
	Full AE model	6	12,980.03	3004	<0.001	1	1	6972.03
	Drop moderation of the mean components	6	14,363.26	3004	1383.23	1	<0.001	8355.26
	Drop moderation of the variance components	4	12,986.58	3006	6.54	3	0.16	6974.58
	Drop all moderation	3	14,373.92	3007	1393.88	4	<0.001	8359.92
HI: Unhealthy dietary intake moderation model								
Full AE model		6	12,995.57	3008	0.06	1	0.80	6979.57
	Drop moderation of the mean components	6	14,376.5	3008	1380.99	1	<0.001	8360.5
	Drop moderation of the variance components	4	13,001.79	3010	6.28	3	0.101	6981.79
	Drop all moderation	3	14,391.42	3011	1395.91	4	<0.001	8369.42

EP: Estimated parameters, IA: Inattention, HI: Hyperactivity-impulsivity, LL: Log Likelihood; df: degree of freedom; AIC: Akaike’s Information Criterion.

## Data Availability

The data that support the findings of this study are available from The Swedish Twin Register (https://ki.se/en/research/the-swedish-twin-registry) upon reasonable request. (accessed on 30 November 2021).

## Supplementary Materials

The following are available online at https://www.mdpi.com/article/10.3390/genes13010047/s1, Table S1. Univariate model-fitting results of bivariate analysis of with 95% confidence intervals and dietary intake; Table S2. Genetic and environmental effects for ADHD symptoms dimensions in different level of dietary intake (with 95%CI).
