# Supplementary material for: Gene-Environment Interactions in Attention-Deficit/Hyperactivity Disorder Symptom Dimensions: The Role of Unhealthy Food Habits
Source: Genes (Basel). 2021 Dec 24;13(1):47. doi: 10.3390/genes13010047 (PMC8774985; doi:10.3390/genes13010047)
Supplement: Supplementary file 1 [file genes-13-00047-s001.zip › genes-1511334-supplementary.pdf]

Supplementary tables

Table S1. Univariate model-fitting results of bivariate analysis of with 95% confidence intervals and dietary intake

	-2LL	df	χ^2	Δdf	*P*-value	AIC
**Inattention**						
ACE model	12842.19	2998	NA	NA	NA	6846.18
**AE model**	**12842.48**	**2999**	**0.29**	**1**	**0.59**	**6844.47**
CE model	12858.67	2999	16.48	1	<0.001	6860.67
E model	12871.01	3000	28.818	2	<0.001	6871.00
**Hyperactivity/Impulsivity**						
ACE model	12995.51	3007	NA	NA	NA	6981.51
**AE model**	**12995.57**	**3008**	**0.06**	**1**	**0.80**	**6979.57**
CE model	13006.94	3008	11.43	1	<0.001	6990.94
E model	13020.52	3009	25.02	2	<0.001	7002.52

IA: Inattention, HI: Hyperactivity-impulsivity, LL: Log Likelihood; df: degree of freedom; AIC: Akaike's Information Criterion. Best-fitting models indicated in bold.

Table S2. Genetic and environmental effects for ADHD symptoms dimensions in different level of dietary intake (with 95%CI)

AE model	Mean	Total variance	Variance (A)	Variance (E)	Proportion of total variance (A)	Proportion of total variance (E)
IA-High-sugar food intake						
Low	2.03 (1.95, 2.12)	3.90(3.59, 4.23)	1.40(0.95, 1.91)	2.49(2.07, 2.95)	0.36(0.25, 0.47)	0.64(0.53, 0.75)
Medium	4.07 (3.90, 4.24)	4.45(4.22, 4.69)	1.81(1.31, 2.12)	2.63(2.40, 2.89)	0.41(0.30, 0.46)	0.59(0.54, 0.65)
High	6.10 (5.84, 6.36)	5.05(4.66, 5.50)	2.28(1.25, 2.88)	2.78(2.33, 3.31)	0.45(0.25, 0.54)	0.55(0.46, 0.65)
IA-Unhealthy dietary intake						
Low	2.03 (1.95, 2.12)	3.95(3.65, 4.28)	1.20(0.78, 1.68)	2.75(2.32, 3.20)	0.30(0.2, 0.41)	0.70(0.59, 0.80)
Medium	4.07 (3.90, 4.24)	4.43(4.19, 4.68)	1.81(1.34, 2.11)	2.61(2.38, 2.87)	0.41(0.31, 0.46)	0.59(0.54, 0.65)
High	6.10 (5.84, 6.36)	5.03(4.63, 5.49)	2.55(1.55, 3.17)	2.48(2.05, 3.00)	0.51(0.31, 0.60)	0.49(0.40, 0.60)
HI-High-sugar food intake						

Low	2.07 (1.99, 2.16)	4.28 (3.95, 4.65)	1.36 (0.91, 1.83)	2.92 (2.51, 3.37)	0.32 (0.22, 0.41)	0.68 (0.59, 0.78)
Medium	4.14 (3.97, 4.32)	4.58 (4.35, 4.83)	1.79 (1.43, 2.10)	2.79 (2.55, 3.07)	0.39 (0.32, 0.45)	0.61 (0.55, 0.67)
High	6.22 (5.96, 6.48)	4.94 (4.55, 5.38)	2.27 (1.68, 2.85)	2.67 (2.24, 3.17)	0.46 (0.35, 0.55)	0.54 (0.45, 0.64)
HI-Unhealthy dietary intake						
Low	2.07 (1.99, 2.16)	4.36 (4.02, 4.74)	1.29 (0.69, 1.79)	3.02 (2.57, 3.52)	0.31 (0.16, 0.40)	0.69 (0.59, 0.79)
Medium	4.15 (3.97, 4.32)	4.55 (4.26, 4.83)	1.79 (1.40, 2.11)	2.76 (2.48, 3.05)	0.39 (0.31, 0.45)	0.61 (0.55, 0.67)
High	6.22 (5.96, 6.48)	4.90 (4.50, 5.36)	2.38 (1.66, 3.02)	2.50 (2.06, 3.04)	0.49 (0.35, 0.58)	0.51 (0.41, 0.62)
